# Comparing and synthesizing quantitative distribution models and qualitative vulnerability assessments to project marine species distributions under climate change

**DOI:** 10.1371/journal.pone.0231595

**Published:** 2020-04-16

**Authors:** Andrew J. Allyn, Michael A. Alexander, Bradley S. Franklin, Felix Massiot-Granier, Andrew J. Pershing, James D. Scott, Katherine E. Mills

**Affiliations:** 1 Gulf of Maine Research Institute, Portland, ME, United States of America; 2 School of Marine Sciences and Technology, University of Massachusetts Amherst, Amherst, MA, United States of America; 3 Cooperative Institute for Research in Environmental Sciences, University of Colorado Boulder, Boulder, CO, United States of America; 4 Physical Sciences Division, National Oceanic and Atmospheric Administration Earth System Research Laboratory, Boulder, CO, United States of America; 5 School of Public Policy, University of California, Riverside, CA, United States of America; 6 Muséum National D’Histoire Naturelle, Paris, France; University of Hamburg, GERMANY

## Abstract

Species distribution shifts are a widely reported biological consequence of climate-driven warming across marine ecosystems, creating ecological and social challenges. To meet these challenges and inform management decisions, we need accurate projections of species distributions. Quantitative species distribution models (SDMs) are routinely used to make these projections, while qualitative climate change vulnerability assessments are becoming more common. We constructed SDMs, compared SDM projections to expectations from a qualitative expert climate change vulnerability assessment, and developed a novel approach for combining the two methods to project the distribution and relative biomass of 49 marine species in the Northeast Shelf Large Marine Ecosystem under a “business as usual” climate change scenario. A forecasting experiment using SDMs highlighted their ability to capture relative biomass patterns fairly well (mean Pearson’s correlation coefficient between predicted and observed biomass = 0.24, range = 0–0.6) and pointed to areas needing improvement, including reducing prediction error and better capturing fine-scale spatial variability. SDM projections suggest the region will undergo considerable biological changes, especially in the Gulf of Maine, where commercially-important groundfish and traditional forage species are expected to decline as coastal fish species and warmer-water forage species historically found in the southern New England/Mid-Atlantic Bight area increase. The SDM projections only occasionally aligned with vulnerability assessment expectations, with agreement more common for species with adult mobility and population growth rates that showed low sensitivity to climate change. Although our blended approach tried to build from the strengths of each method, it had no noticeable improvement in predictive ability over SDMs. This work rigorously evaluates the predictive ability of SDMs, quantifies expected species distribution shifts under future climate conditions, and tests a new approach for integrating SDMs and vulnerability assessments to help address the complex challenges arising from climate-driven species distribution shifts.

## Introduction

Species distributions are shifting in response to warming ocean temperatures, triggering complex ecological, conservation and management challenges. Temperature changes can alter growth, survival and productivity rates in marine organisms, leading to shifts in local distribution and abundance [1–4]. Physiological and ecological constraints mediate the ability of individual species to track preferred temperatures, resulting in unequal responses to climate change [4–7]. These differential responses can create novel biological communities [8–10], in turn, altering the structure and function of marine ecosystems [2,4,11]. Such complex species responses and community transformations test our traditional conservation and management systems [11–14].

Accurate projections of species distribution and abundance under future climate conditions are needed to meet the challenges arising from species distribution shifts [4,14,15]. Species distribution models (SDMs) are one of the most popular quantitative tools for describing, understanding and projecting species distributions [16–18]. Generally, SDMs correlate species occurrence data with environmental variables through linear, additive or non-linear relationships [16,19]. They have many advantages, including their ability to accurately make projections for different scenarios across various spatial and temporal scales [20–22]. They have been criticized, however, for lacking mechanistic relationships, ignoring biological processes, and for their limited transferability when applied to new geographical areas [16,21,22].

Trait-based climate vulnerability assessments provide an alternative tool to assess potential climate change effects on species and are becoming increasingly popular in marine ecosystems (e.g., [23,24]). Rather than trying to develop spatially-explicit, climate-driven projections of species distribution and abundance, in a climate vulnerability assessment experts rank species vulnerability to climate change based on exposure, sensitivity and adaptive capacity. Exposure estimates the expected characteristics, magnitude and rate of climate change a species will experience; sensitivity characterizes a species’ susceptibility to these changes given its intrinsic traits and life history characteristics; and adaptive capacity represents a species’ ability to cope with these changes by adjusting its behavior, physiology or life history [22,25,26]. Vulnerability assessments can identify and prioritize conservation and management actions relatively quickly for a large number of species, while exploring mechanisms between climate changes and species responses [21–23,27]. However, they only produce relative vulnerability rankings, lack spatial granularity, and can be difficult to validate [22].

Given the comparative advantage of each method, there may be opportunities to combine SDMs with vulnerability assessments to produce more accurate species distribution and abundance projections. For example, SDMs produce granular, spatially-explicit projections of relative biomass that are useful for regional managers and communities making decisions and planning for potential system changes. In contrast, qualitative vulnerability assessments are usually conducted at large scales, and cannot convey the severity of impact in a local area or the degree to which a particular area will follow broad-scale patterns. However, qualitative expert assessments provide information on many processes that SDMs do not account for that influence the distribution and abundance of a species, such as species’ sensitivity based on biological and ecological traits like dispersal, mobility, and prey specificity [21,22]. Therefore, integrating climate vulnerability and species distribution projections may help account for expected large-scale changes in population processes while also producing results that can be relevant to local spatial scales.

Most existing combined approaches require an explicit connection between the quantitative and qualitative efforts. For instance, SDM projections have been used as a guide to help experts when completing vulnerability assessments [28–30]. Additionally, expert knowledge has been incorporated to refine SDM projections [31–33] and to characterize prior distributions of SDM parameters [34–36]. Coupling the two efforts more tightly in a “semi-quantitative” approach, Sortini et al. [37] summarized results from SDM projections and then included these summaries as exposure metrics while assessing species vulnerability to ocean warming. Although the application of combined approaches to project marine species distributions is relatively new, they have reduced uncertainty for climate-driven projections of other natural phenomena (e.g., Antarctic sea ice responses to climate change [38,39]), highlighting their potential to provide more accurate and robust marine species distribution projections.

In the combined approaches described above, efforts are linked as SDMs are incorporated into the assessment process or experts inform the fitting of the SDMs, but this might not always be possible. Occasionally, quantitative modeling efforts and qualitative assessments are completed independently (e.g., Northeast Shelf Large Marine Ecosystem, USA [23,40] and southern Benguela ecosystem, Africa [24,41]). This hinders our ability to combine methods. Instead of one “combined” model, we are left with two, independent pieces of information (numeric quantitative model projections and ranked qualitative expert assessments) with no methodological way of comparing or synthesizing them into a “best estimate” of how species will respond to climate change. In these situations, our understanding of climate-driven species distribution and abundance changes and our capacity to adapt to these changes could be improved by answering three questions. First, can we make comparisons between the methods to learn more about their strengths and weaknesses? Second, are there traits or life history characteristics shared across species that determine when quantitative projections and qualitative expectations match? Finally, can we develop approaches that combine the two independent methods to produce more accurate projections of species distribution and abundance under future climate change?

The Northeast Shelf Large Marine Ecosystem (NELME) provides an ideal testing ground for asking these questions. From 1982 through 2018, the region’s surface waters warmed faster than the vast majority of the world’s oceans [42–44], and it is projected to warm rapidly in the future [45,46]. Researchers have also documented temperature-driven movements of marine species to higher latitudes and to deeper depths [47,48], and projections indicate distribution changes will continue [40,49]. Additionally, the NELME was one of the first U.S. marine regions to complete an expert climate vulnerability assessment for ecologically and economically important marine fish and invertebrate species [23].

In this study, we synthesized quantitative SDMs and qualitative expert climate change vulnerability assessments to project marine species distributions under climate change in the NELME. We first developed and validated quantitative SDMs for a suite of marine species and then used the fitted models to project species distributions to mid-century (2055) given sea surface temperature conditions expected under a “business as usual” climate scenario. Next, we compared these SDM projections with the expectations from an independent climate change vulnerability assessment [23] to identify common patterns and determine whether these patterns could be attributed to species traits or characteristics. Finally, we developed and evaluated a novel approach for combining the quantitative SDMs and expert assessments that supports spatially-explicit species distribution and abundance projections. This integrated approach may provide new information and insights to help meet the complex challenges triggered by climate-driven species distribution shifts.

## Methods

### Data collection

#### Quantitative data for species distribution models

**Fig 1:**
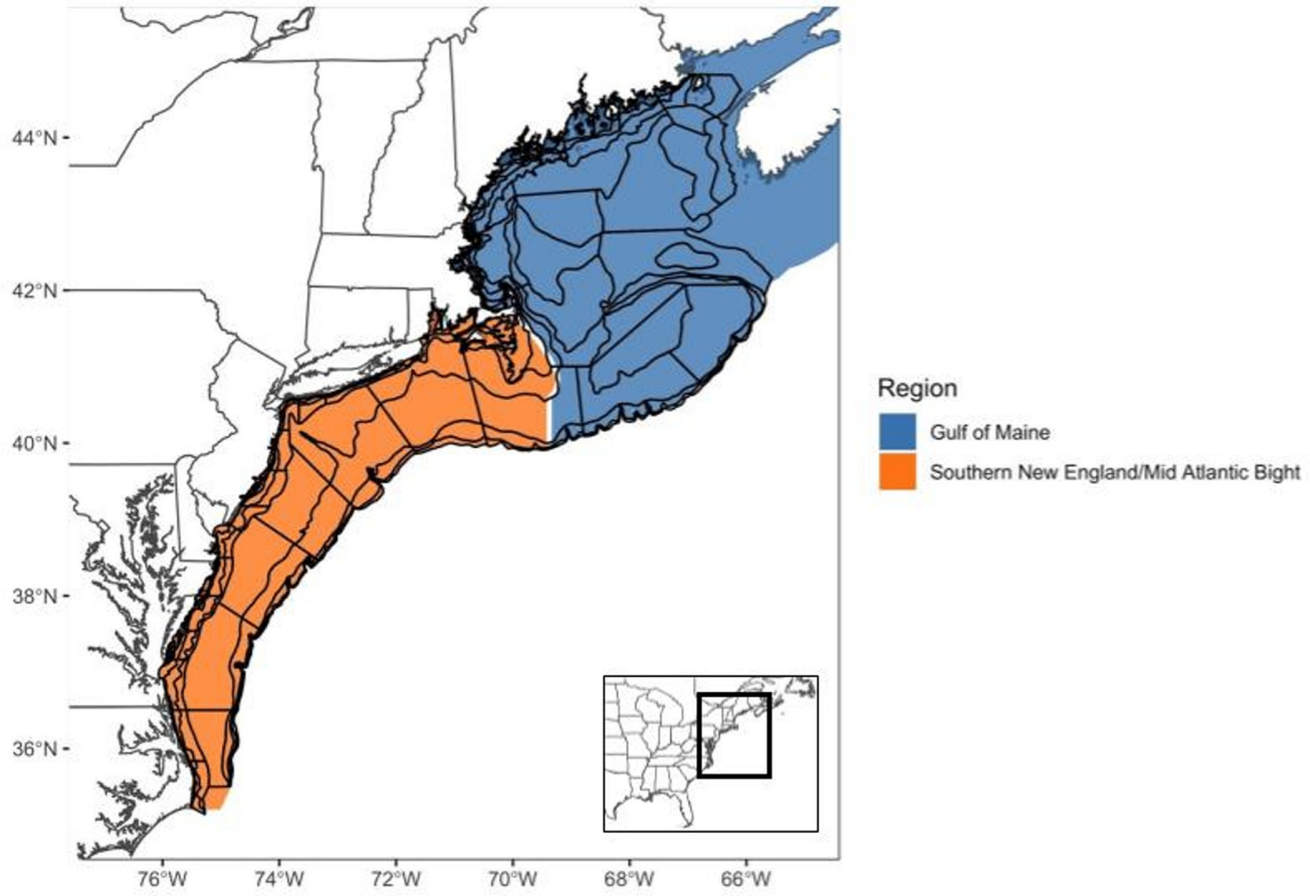
The Northeast Shelf Large Marine Ecosystem study area [53]. Polygons within the Northeast Shelf Large Marine Ecosystem designate the habitat strata used to create a stratified random bottom trawl survey, conducted by the NOAA Northeast Fisheries Science Center. Within the Northeast Shelf Large Marine Ecosystem, there are two relatively distinct biogeographic regions: the Gulf of Maine (blue shaded habitat strata polygons) and the southern New England/Mid Atlantic Bight (orange shaded habitat strata polygons).

*Species biomass*. We gathered species biomass data from spring and fall bottom trawl surveys conducted since 1968 by the NOAA Northeast Fisheries Science Center (NEFSC) [50–52]. These surveys cover an area from Cape Hatteras, North Carolina to the Gulf of Maine, including Georges Bank (Fig 1). A stratified random sampling design divides the region into strata based on depth, bottom habitat type and latitude. During a given survey, stations are randomly selected within each stratum proportional to the stratum area, with a minimum of two successful stations required in each stratum [52]. At each station, a bottom trawl net is towed along consistent depth contours for a set time and speed. The catch is then sorted to species, counted and weighed.

To standardize fisheries survey data, we first excluded data from non-representative tows (i.e., short tow durations or tows which had substantial gear problems). In addition, we applied standard calibration factors [54–56] to adjust species biomass estimates based on vessel, gear, and door changes that have occurred over the duration of the survey. While this produces standardized biomass estimates, the bottom trawl gear inherently samples certain species better than others and will not capture all individuals encountered (i.e., catchability is less than 1). Therefore, biomass estimates are considered on a relative scale, with comparisons limited to within, not among, species.

We reduced the full NEFSC bottom trawl survey dataset to include focal species that were evaluated by Hare et al. [23] in a climate vulnerability assessment of fish and invertebrate species in the NELME. The study assessed 84 total species, which Hare et al. [23] grouped into six categories, resulting in 14 coastal fish, 10 diadromous fish, 12 elasmobranchs, 19 groundfish, 19 benthic invertebrates, and 10 pelagic fish and cephalopods (S1 Table).

*Observation period environmental characteristics*. To understand the environmental drivers of species relative biomass patterns, we gathered measurements of static and dynamic ecosystem variables associated with each completed tow. We used depth and seasonal sea surface temperature (SST) to characterize local ecosystem conditions, as they are well known to influence the distribution and abundance of marine fish and invertebrates. Habitat survey stratum is also sometimes included as a factor in marine fish SDMs (e.g., 40). We elected not to include this factor variable, assuming that species may move across habitat strata boundaries to find similar depth/temperature habitats and to reduce overall model complexity as there are 111 unique habitat strata. We chose a seasonal scale for the SST variable to target the seasonal temperature signal. During a given tow, the *in situ* or daily temperature may be warmer or colder than the seasonal average, yet we expect the observed seasonal distribution and relative biomass of a species is better reflected by average temperatures experienced over multiple weeks and months. We used SST instead of bottom temperature in our models since we were interested in modeling a suite of species, which included coastal, diadromous, and pelagic fish species that regularly use the entire water column. Additionally, SST is available in more global climate model simulations than temperature at depth [46].

We collected depth and SST data from online NOAA resources. Depth data were downloaded from the NOAA ETOPO1 Global Relief Model [57], which integrates topographic elevation measurements and ocean bathymetric measurements with a 1 arc-minute resolution. The depth at specific tow locations was extracted using bilinear interpolation of depth values from four neighboring cells with the R *raster* package v3.0–7 [58]. Daily SST data were gathered from the NOAA Optimum Interpolation Sea Surface Temperature (OISST) dataset, with a spatial resolution of 0.25 degrees [59,60]. We first collected the full OISST time series, which spanned from 1982 to present, at each of the tow locations and then averaged daily temperature records over a season. For tows completed during the spring, we averaged all SST values between March and May, and for tows in the fall we averaged SST values between September and November. These months capture the timespan when each seasonal survey was completed.

*Projected environmental characteristics*. We used an ensemble of climate model simulations to estimate future SSTs, and then used these temperatures to project species distribution and relative biomass. The ensemble included 27 climate model members that are part of the Climate Model Intercomparison Project (CMIP-5, http://cmip-pcmdi.llnl.gov/cmip5/data_portal.html; S2 Table) archive. Each ensemble member used observed greenhouse gas concentrations for 1976–2005 and the Representative Concentration Pathway (RCP) 8.5 (“business as usual”) scenario for greenhouse gas emissions over the remainder of the 21st century. Given varying spatial resolutions across the members in the ensemble, all projections were rescaled to a common 1x1 degree longitude/latitude grid. The varying spatial resolution of each member also created gaps in SST outputs for cells located along the continental border of the NELME. To estimate projected temperatures in these coastal cells, we interpolated temperature values by taking the average SST of all bordering cells.

From these model outputs, we estimated future SSTs. For each of the 27 members, we first calculated a baseline monthly climatology by averaging estimated temperatures at each grid cell for each month from 1982 through 2011. We then calculated year-month temperature anomalies for 1982–2055 from the 1982–2011 climate model ensemble member’s climatology. After calculating these anomalies, we added them to the 1982–2011 climatology calculated from the observed OISST data. This yielded downscaled year-month temperature estimates from 1982 to 2055, where the resolution of the downscaled estimates matched the resolution of the OISST data (0.25 degree grid cells). Adding the anomalies to the observed OISST climatology rather than the climatology from the ensemble member helped account for the tendency of most climate models to project the Gulf Stream current continuing northward along the coast rather than turning eastward around Cape Hatteras, NC, which leads to a “warm bias” of Northeast U.S. continental shelf ocean water temperatures [45,61]. After removing the warm bias, we determined the monthly ensemble mean, 5th (second coldest model temperature) and 95th (second warmest model temperature) percentiles using estimated temperatures from the 27 members (S1 Doc). Finally, we calculated 2055 fall and spring seasonal average temperatures for each grid cell by averaging March, April and May temperatures for spring and September, October and November temperatures for fall.

#### Qualitative data from expert climate vulnerability assessment

Qualitative data about expected climate change effects on species throughout the NELME were taken from expert scoring data, originally collected as part of the National Marine Fisheries Service’s Northeast U. S. species vulnerability assessment (NEVA, 23). During this assessment, experts ranked the climate exposure, climate sensitivity and the directional effect for 84 species inhabiting the region. The experts were instructed to make their rankings based on projections of climate change effects under the RCP8.5 scenario. Climate exposure measured the severity of change in the physical environment (12 factors), while sensitivity measured biological and ecological traits that may limit or enhance a species’ ability to respond to changes in the physical environment (12 factors). The anticipated directional effect measured whether climate change would have a negative, neutral or positive effect on a species’ productivity.

We used the overall sensitivity and directional effect rankings, as well as the disaggregated voting data in our analysis. Details on the suite of logic rules and weighting schemes used to calculate overall species rankings can be found in Hare et al. [23]. We focus on describing the disaggregated voting data, as it plays an important role in our combined modeling approach that synthesizes quantitative SDM and expert assessment projections. To rate climate sensitivity, five experts were given five tallies to score each of the exposure or sensitivity attributes. Experts placed their five tallies into four bins (low, moderate, high, very high), and the distribution of tallies across bins depended on how certain an expert was on the degree of sensitivity for a given factor. For example, when evaluating a species with very well-known limited dispersal ability, an expert may place all five tallies in the ‘very high’ bin for the dispersal ability sensitivity attribute. Given this voting scheme, there were 25 votes per species per sensitivity attribute. For each species, we summed across attributes by bin, resulting in a dataset that could be described as a random outcome from a multinomial distribution with four bins. Scoring for the directional effect was similar to the scoring of sensitivity attributes. However, three experts were only provided four tallies to distribute across three bins (negative, neutral, positive), creating a dataset that could be described using a multinomial distribution with three bins.

### Data analysis

#### Species distribution model fitting and validation

To model current and project future species distributions, we used a two-stage delta generalized additive model (GAM) [62]. This modeling approach has been widely used in other marine fish distribution modeling studies [40,48,49] and has several advantages. First, the two stage approach models presence/absence and then models the log positive biomass observations [63–65], and this structure accommodates situations where the number of absence observations exceeds those expected from traditional “count” distributions (e.g., Poisson, tweedie). Second, the additive modeling framework requires no *a priori* assumptions about the functional relationships between the response (species presence/absence and biomass) and predictor variables, allowing for non-linear relationships [62,66]. For each species, we fit seasonal delta log-normal GAMs with the *gam* function in the R *mgcv* package v1.8–29 [62,67]. We used penalized cubic regression splines for depth and SST smooth terms and a default of 10 knots. Additionally, we used the function’s built in “select” option to remove depth or temperature variables if they had no influence on either the presence/absence or logged positive biomass response.

We fitted models to training data from 1982–2011 and then validated models to testing data from 2011–2015. To validate each distribution model, we first calculated the area under the receiver operating curve (AUC) statistic for the presence/absence model stage. The AUC statistic is a measure of a model’s classification ability and determines whether testing data presences correspond with higher model predicted probabilities than testing data absences. As such, AUC is only concerned with relative model predicted probabilities [68]. To provide a more rigorous assessment of model prediction accuracy that incorporates the actual predicted values, we also plotted Taylor diagrams [69] for overall biomass predictions (i.e., probability of presence * log positive biomass). Taylor diagrams display three measures of model validation: the correlation between model predictions and observations, the model predictions centered root mean square error (RMSE), and the ratio between the standard deviation of model predictions to the standard deviation of observations. In combination, these diagrams then provide insight into the agreement between model predictions and observations (correlation coefficient and RMSE) as well as if the model is producing predictions that exhibit similar spatial variability as observational data (standard deviation ratios). By normalizing RMSE and standard deviation ratio statistics, Taylor diagrams can also plot results for different species on one diagram.

#### Species distribution model projections

To project species distribution and biomass to spring and fall 2055, we used mean, 5^th^ and 95^th^ percentile SSTs from the RCP 8.5 climate model ensemble. Projections were first made with the presence/absence model and then the logged positive biomass model. Overall relative biomass was then calculated by multiplying the projected probability of presence by the exponentiated log positive biomass projections. We explored changes within specific regions of the NELME by summarizing average projected relative biomass changes within the Gulf of Maine (GoM) area and the southern New England/Mid-Atlantic Bight (SNE-MAB) area (Fig 1). These two regions are divided by a well-known physiographic break just south of Cape Cod, Massachusetts, which provides an interesting opportunity to examine if the distribution and relative biomass of a species is changing similarly across the whole shelf. For a few species, unique region-season combinations produced projected biomass increases that were very high [Atlantic croaker (*Micropogonias undulates*), Northern kingfish (*Menticirrhus saxatillis*), Spot (*Leiostomus xanthurus*), weakfish (*Cynoscion regalis*), horseshoe crab (*Lumulus polyphemus*) and clearnose skate (*Raja eglanteria*)], usually resulting from very low biomass during the baseline period. To make visual comparisons among species and across potential climate conditions easier, we capped these projected changes at 500%.

#### Comparing species distribution model projections and expert climate vulnerability assessments

To compare SDM results to the expert climate vulnerabiity assessment rankings, we reclassified the continuous numeric SDM seasonal projections into climate sensitivity and directional effect ranks. To determine the climate sensitivity ranking from the quantitative distribution model projections, we followed Crossman et al. [70] and calculated a sensitivity weight (SensWeight) across all grid cells (*m*), where

$$SensWeight=\frac{\sum_{k=1}^{m}\left|Baseline\;predited\;relative\;biomass\;–\;Future\;projected\;relative\;biomass\right|}{\sum_{k=1}^{m}Future\;projected\;relative\;biomass}$$

For each species, we averaged the sensitivity weights for fall and spring. We then assigned these values to ranks depending on the percentile of a species’ sensitivity weight relative to all species, where low sensitivity = 0–25%, moderate sensitivity = 25–50%, high sensitivity = 50–75% and very high sensitivity = 75–100%.

To translate model projections into directional effect bins, we determined the projected percent change from the seasonal average baseline and future projected relative biomass across all cells in the NELME and then calculated the average projected percent change between the two seasons. These average projected percent relative biomass change values were used to characterize the directional effect of each species, such that changes < -25% were a negative directional effect, -25% to 25% were a neutral directional effect, and >25% change were a positive directional effect.

After categorizing the SDM projections into sensitivity and directional effect ranks to correspond with the rankings used in the expert assessment, we investigated if certain sensitivity attributes might explain when quantitative distribution model rankings agreed or disagreed with the expert assessment rankings. To do this, we only kept species with SDM AUC values > = 0.7 and ones that had at least moderately certain expert sensitivity and directional effect assessment certainties. Then we fitted a random forest model using the R *randomForest* package v4.6–14 [71] to classify whether the two rankings matched, with the voting data ranks (low, moderate, high or very high) for each of the 12 sensitivity attributes included as independent variables and ‘match’ or ‘mismatch’ as the response variable.

#### Combining species distribution model and expert assessment projections

We combined the quantitative SDM and qualitative expert assessment projections using Bayes’ Theorem, which states that given two events (A and B) the conditional probability of event A given event B (i.e., posterior) is

P(A|B)≈P(B|A)*P(A)

In our approach, we were interested in determining the probability of observing the quantitative SDM (event A) given the qualitative expert assessment sensitivity and directional effect rankings (event B). Calculating the posterior probability then was a matter of calculating the likelihood of the qualitative expert assessment rankings given the quantitative distribution model, P(B|A) and the prior probability of the quantitative distribution model, P(A).

To implement this calculation, we first isolated the log positive biomass stage of each fitted seasonal GAM. We did this because experts were instructed to assess how sensitivity attributes and directional effects mapped to the abundance and productivity of a fish stock [22], not the presence or absence of a given species. From the fitted model object, we then drew a sample (n = 1000) of the model parameters from a multivariate normal distribution using the *rmvnorm* function in the *mvtnorm* package v1.0–11 [72,73], with mean equal to the fitted parameter values and covariance matrix equal to the variance-covariance of the fitted model. A given set of model parameters from this sample represented one candidate model. Notably, this procedure departs from a true Bayesian implementation of the combined method, as we fixed the candidate distribution models, rather than using a sampling algorithm (e.g., Monte Carlo Markov Chain). We decided this approach ensured that the candidate model generation process maintained plausible model parameters and covariances among parameters as the relationship between species relative biomass and each predictor variable in the distribution model could be described by up to 10 different parameters, given the use of additive, smooth functions. For each of the candidate models, we then used its parameters to predict the relative seasonal log biomass during the baseline period (2011–2015) and the future period (2055) for each species across the entire NES LME. Exponentiating these predictions and multiplying them by the probability of presence from the presence/absence stage of the model, which remained constant for all candidate models, yielded spatially-explicit relative biomass predictions for the baseline and future periods.

**Fig 2:**
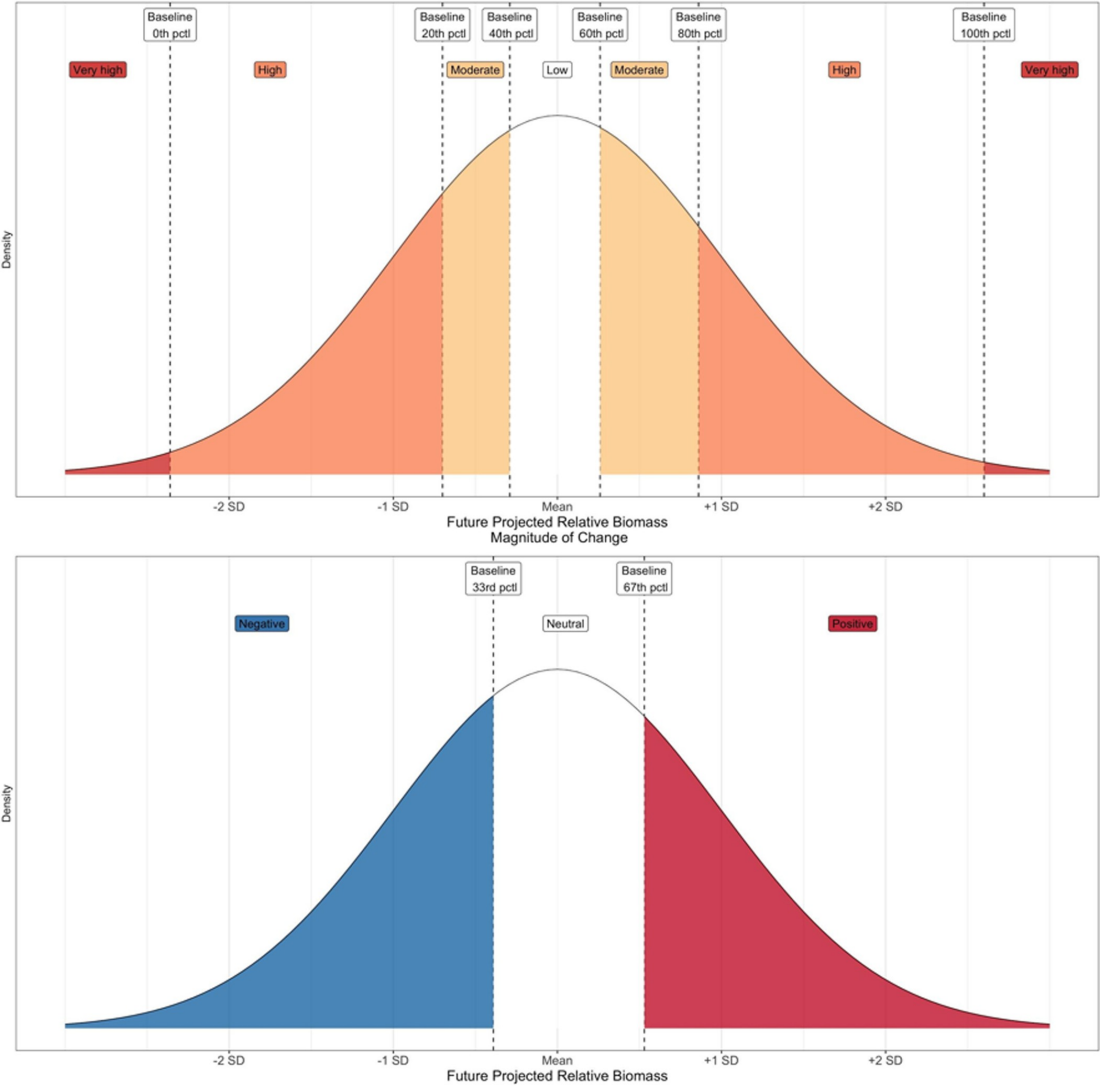
Calculating sensitivity and directional effect bin probabilities. These figures depict how we calculated sensitivity bin probabilities (top figure) and directional effect in probabilities (bottom figure) using baseline predicted relative biomass percentiles and the mean and standard deviation for the future projected relative biomass. For the sensitivity component, we were specifically focusing on the absolute magnitude of change, while for the directional effect component we accounted for the relative change. Color coded shaded regions are used to highlight the total probability of being in each sensitivity bin (very high, high, moderate, low) and directional effect bin (negative, neutral, positive).

After making these predictions, we evaluated how likely the sensitivity votes were given the magnitude of change expected from the candidate distribution model, and how likely the directional effect votes were given the directional change expected from the candidate distribution model. For the first component (i.e., likelihood of expert sensitivity voting given the magnitude of change in SDM biomass), we took the relative biomass predictions during the baseline period for every cell within the NES LME and calculated the 0, 20, 40, 60, 80 and 100th percentiles. For the future period, we calculated the mean (F_mn_) and standard deviation (F_SD_) from the candidate model projections. We then used the R *stats* package *pnorm* function [74] to calculate the cumulative probability of an observation being less than or equal to each of the baseline percentile values, given a normal distribution with mean = F_mn_ and standard deviation = F_SD_. We aligned these values to the sensitivity bins to obtain the total probability of being in the very high, high, moderate or low sensitivity bins (Fig 2).

This binning approach implies that a species’ sensitivity can be represented in a statistical distribution such that a “very high” sensitivity means that there is a greater chance of observing extreme future values that would fall in the tails of the distribution, while a “low” sensitivity means that there will be a greater chance of observing future values near the middle of the distribution. Next, we calculated the total number of votes across all sensitivity attributes for each of the low, moderate, high and very high ranks. Finally, we determined the probability of observing the sensitivity rank votes given the per-bin probabilities calculated from the distribution model with the *dmultinom* function in the *stats* package [74].

We used a similar procedure to calculate the second component (i.e., likelihood of the expert directional effect voting given the directional change predicted by the candidate SDM). However, instead of calculating six percentile values like we did for sensitivity, we only needed to calculate two: the 33rd and the 67th percentile. We again used the *pnorm* function to get the cumulative probability at each of the percentile values and then determined the probability a value was less than 33rd percentile (negative), between the 33rd and 67th percentile (neutral), or greater than the 67th percentile (positive). Following our process for the sensitivity binning, we assumed that the projected biomass for a “negative” species would fall within the lower third of the distribution, whereas a “positive” species would have projected biomass values within the upper third of the distribution (Fig 2). Again, we used the *dmultinom* function to determine the probability of observing the species directional effect votes given the per-bin directional effect probabilities. The overall likelihood of the qualitative votes (both the sensitivity and directional effect components) given the distribution model was then calculated by adding two independent log probability values: (1) the log probability of observing the sensitivity rank votes given the sensitivity per-bin probabilities and (2) the log probability of observing the directional effect votes given the directional effect per-bin probabilities.

The next step in the combination process required calculating the probability of a given distribution model from the candidate model samples (i.e., the prior). Since these candidate models were all pulled from a multivariate normal distribution, we used the *dmvnorm* function in the *mvtnorm* package v1.0–11 [72,73] to calculate the logged probability of the selected candidate model values given a multivariate normal distribution with mean equal to the fitted parameter values and covariance matrix equal to the variance-covariance of the fitted model.

Finally, we calculated the posterior probability of observing the candidate model parameters given the expert assessments by multiplying the logged probabilities for the likelihood and the prior. After completing this process for all candidate model parameters, we could then determine which candidate model was most likely. We used the most likely model to evaluate whether the combined model produced more accurate or robust predictions to the baseline period than the quantitative SDM. We then investigated the difference in projected absolute magnitude of change in relative biomass and mean change in relative biomass between the two models. We also plotted the fitted smooth functions for each model to see what effect the combined model had on these relationships.

## Results

### Species distribution model

#### Model validation

Of the 82 species included in the qualitative climate vulnerability expert assessment by Hare et al. [23], we were able to fit SDMs to 49 species that had at least “good” ability to correctly classify species presence/absence in both seasons (AUC > = 0.7, S1 Table). Models performed relatively well for most groups of species, especially groundfish, as 17/19 groundfish species models met this standard. The major exceptions were species in the diadromous fish functional group, where only 2 of 10 species had models with AUC > = 0.7. This variability in model performance across the different functional groups is not surprising, given that the species data are from a bottom trawl survey, which best targets groundfish species. All subsequent results we report are for species that met this AUC threshold.

**Fig 3:**
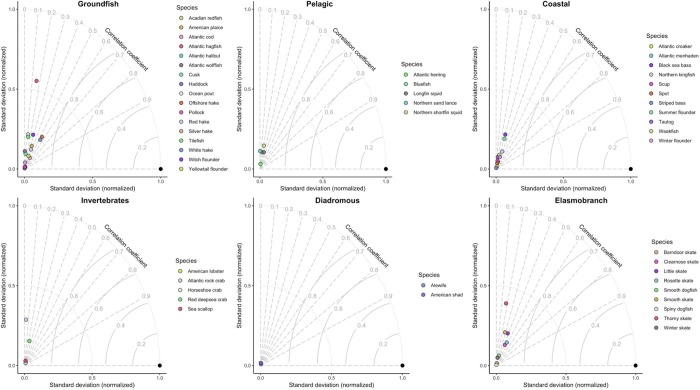
Taylor diagram of fall model predicted biomass compared to 2011–2015 observed biomass. The Taylor diagram provides a succinct picture of a model’s predictive ability. For each Taylor diagram, there is a reference point (shown by the black filled circle) at [1,0]. This point indicates a model with perfect correlation between observations and predictions, no error, and the correct level of spatial variability. The relative strength of different models for different species is assessed by where the species-model point falls on the Taylor diagram, where correlation between observations and model predictions increases moving along the correlation coefficient arcs, reaching perfect correlation at the horizontal line with y = 0, model root mean square error decreases proportionally with the radial distance of each point to the reference point at [1,0], and agreement between observed variability and predicted variability increases with radial distances from the origin [0,0].

**Fig 4:**
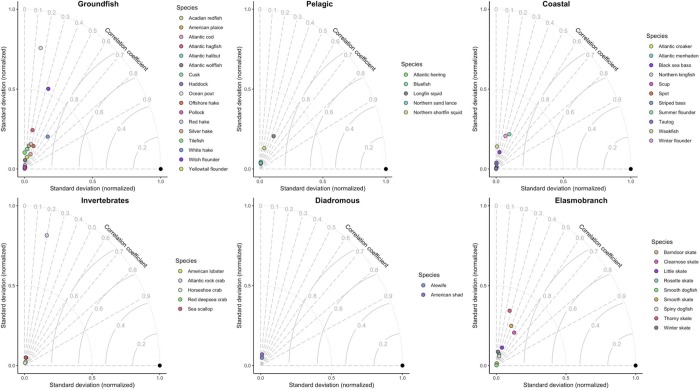
Taylor diagram of spring model predicted biomass compared to 2011–2015 observed biomass. The Taylor diagram provides a succinct picture of a model’s predictive ability and a detailed description of its features are provided in the caption for Fig 3.

Taylor diagrams provided a more rigorous assessment of the model’s ability to accurately predict species biomass and capture the correct level of variability (Figs 3 and 4). In general, correlations between model predicted biomass and observed biomass were greatest for groundfish, elasmobranch and coastal fish species, while models for pelagic, diadromous and invertebrate species struggled to reach correlation coefficients greater than 0.3. Across all species models, there was a tendency for the models to underrepresent expected variability in biomass predictions (i.e., to smooth over expected spatial variability), indicated by the clustering of species models near the origin of each Taylor diagram. The large distances between the reference point and the species models also indicated that RMSE values were relatively high for most species, but slightly less for groundfish, coastal and elasmobranch species.

#### Projected changes across the Northeast Shelf Large Marine Ecosystem

**Fig 5:**
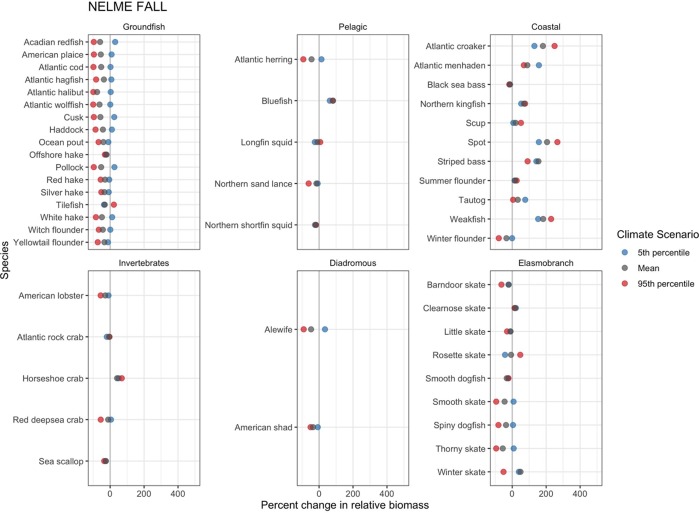
Fall projected shelf-wide percent change in relative species biomass. Projected species relative biomass changes given the 5th percentile, mean, and 95th percentile CMIP5 RCP 8.5 climate model ensemble projected sea surface temperatures.

Across the NELME and under mean projected 2055 SSTs, the relative biomass of most species is expected to decline in the fall (Fig 5). These declines are most ubiquitous among species in the groundfish group. With the exception of tilefish (*Lopholatilus chamaeleonticeps*) and offshore hake (*Merluccius albidus*), all groundfish species also decline more severely when projections were made using 95^th^ percentile SSTs of the climate model ensemble. Interestingly, many of these same species show projected increases in relative biomass, albeit at fairly low levels, under the 5^th^ percentile SST conditions, which represents cooler temperatures than under baseline conditions. Other notable species following this same general pattern include Atlantic herring (*Clupea harengus*) and northern sand lance (*Ammodytes dubius*) (pelagic), winter flounder (*Psuedopleuronectes americanus*) (coastal), American lobster (*Homarus americanus*) and deep-sea red crab (*Chaceon quinquedens*) (invertebrates), spiny dogfish (*Squalus acanthias*), smooth (*Malacoraja senta*) and thorny skates (*Amblyraja radiata*) (elasmobranchs).

Coastal fishes represent a major exception to the pattern of projected declines in the fall. Within this group, the relative biomass for the vast majority (9/11) of species is projected to increase under mean future SSTs (Fig 5). Unlike the dominant groundfish pattern, coastal species show considerable variability among the three warming conditions. For instance, many of the species exhibit greatest relative biomass projections under 95^th^ percentile SST conditions. However, Atlantic menhaden (*Brevoortia tyrannus*), striped bass (*Morone saxatillis*) and tautog (*Tautoga onitis*), all have their smallest projected increases under these temperatures.

**Fig 6:**
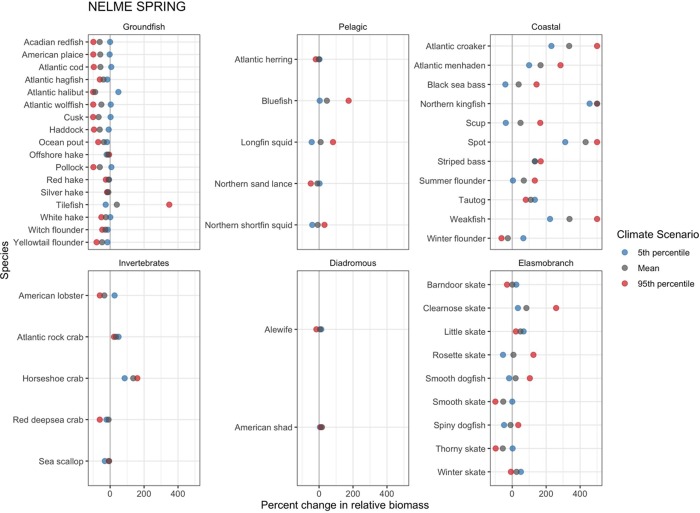
Spring projected shelf-wide percent change in relative species biomass. Projected species relative biomass changes given the 5th percentile, mean, and 95th percentile CMIP5 RCP 8.5 climate model ensemble projected sea surface temperatures. Positive percent increases were capped to 500% for four coastal fish species (Atlantic croaker, Northern kingfish, spot, and weakfish) to facilitate visualizing and comparing changes across species and climate model ensemble projected sea surface temperature scenarios.

In the spring, most species follow similar patterns as exhibited during the fall (Fig 6), with the exception of American lobster, smooth (*Mustelus canis*) and spiny dogfish. Although patterns are similar, many species show considerable differences in the magnitude of projected changes between the two seasons, including Atlantic croaker, spot and Northern kingfish under all warming conditions and tilefish, weakfish, longfin squid, Atlantic menhaden and clearnose skate under 95^th^ percentile SSTs. Overall, the most noticeable difference between the two seasons arises in patterns of variability among the climate model ensemble 5^th^ percentile, mean and 95^th^ percentile SSTs. For some species groups, relative biomass appears to be more sensitive to SST in the spring versus the fall, resulting in greater differences in relative biomass changes across the warming conditions. This increasing variability is particularly evident for species in the pelagic, coastal and elasmobranch functional groups.

#### GoM vs. SNE-MAB changes

**Fig 7:**
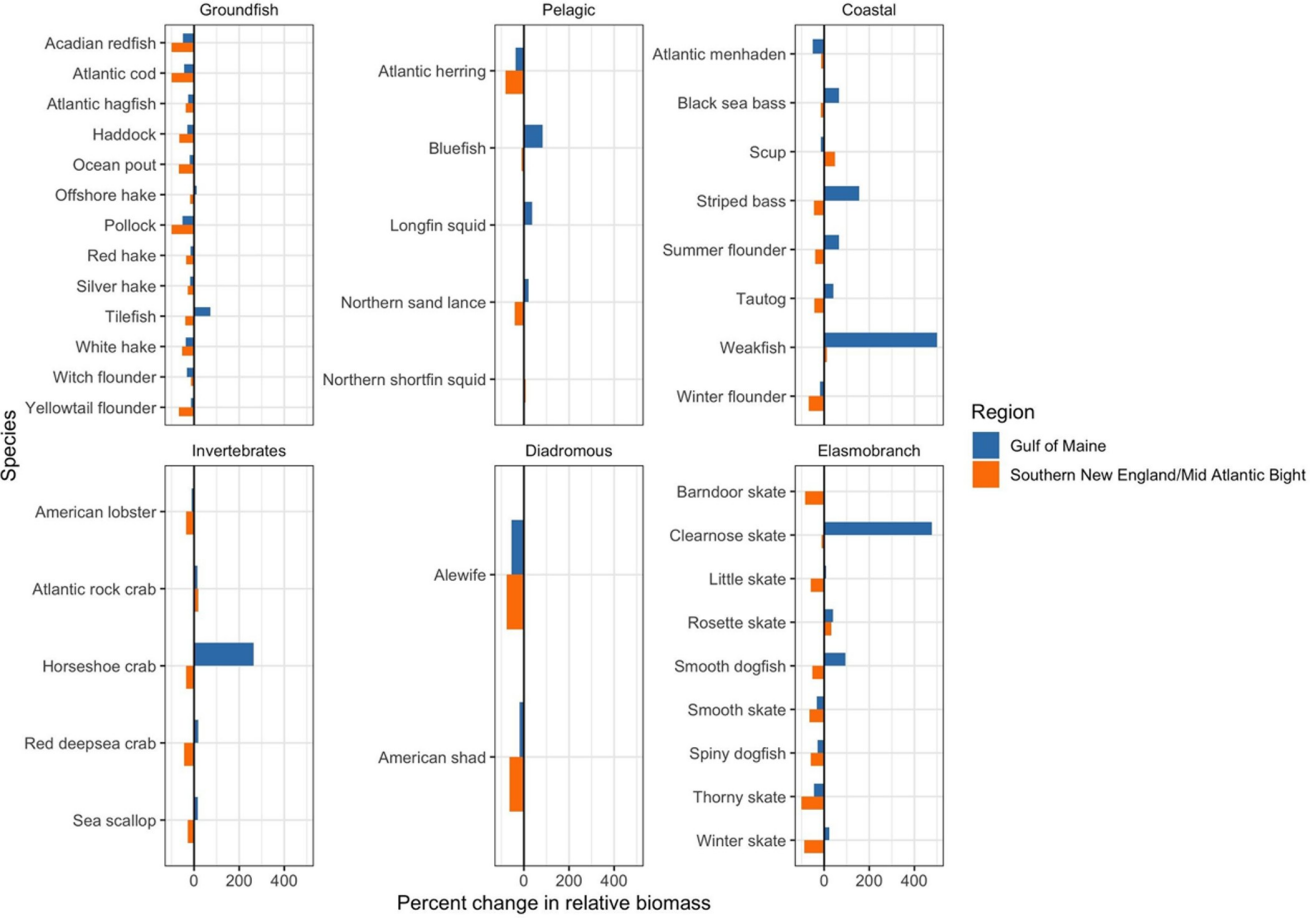
Fall regional projected percent changes in relative biomass by species functional group. Projected species relative biomass changes given the mean CMIP5 RCP 8.5 climate model ensemble projected sea surface temperatures. Positive percent increases were capped to 500% for weakfish to facilitate visualizing and comparing changes across species and climate model ensemble projected sea surface temperature conditions.

In the fall, most species, especially groundfish and elasmobranchs, show declines in both regions, with SNE-MAB declines expected to be more severe than declines in the GoM (Fig 7). The only groundfish species expected to increase in relative biomass is tilefish in the GoM. Divergent projections between the two subregions arise predominantly for coastal fish species. Black sea bass (*Centropristis striata*), striped bass, tautog and summer flounder (*Paralichthys dentatus*) are all expected to decline in the SNE-MAB and increase in the GoM. Scup (*Stenotomus chrysops*), also a coastal fish species, is the only species that is projected to increase in the SNE-MAB region and decrease in the GoM.

**Fig 8:**
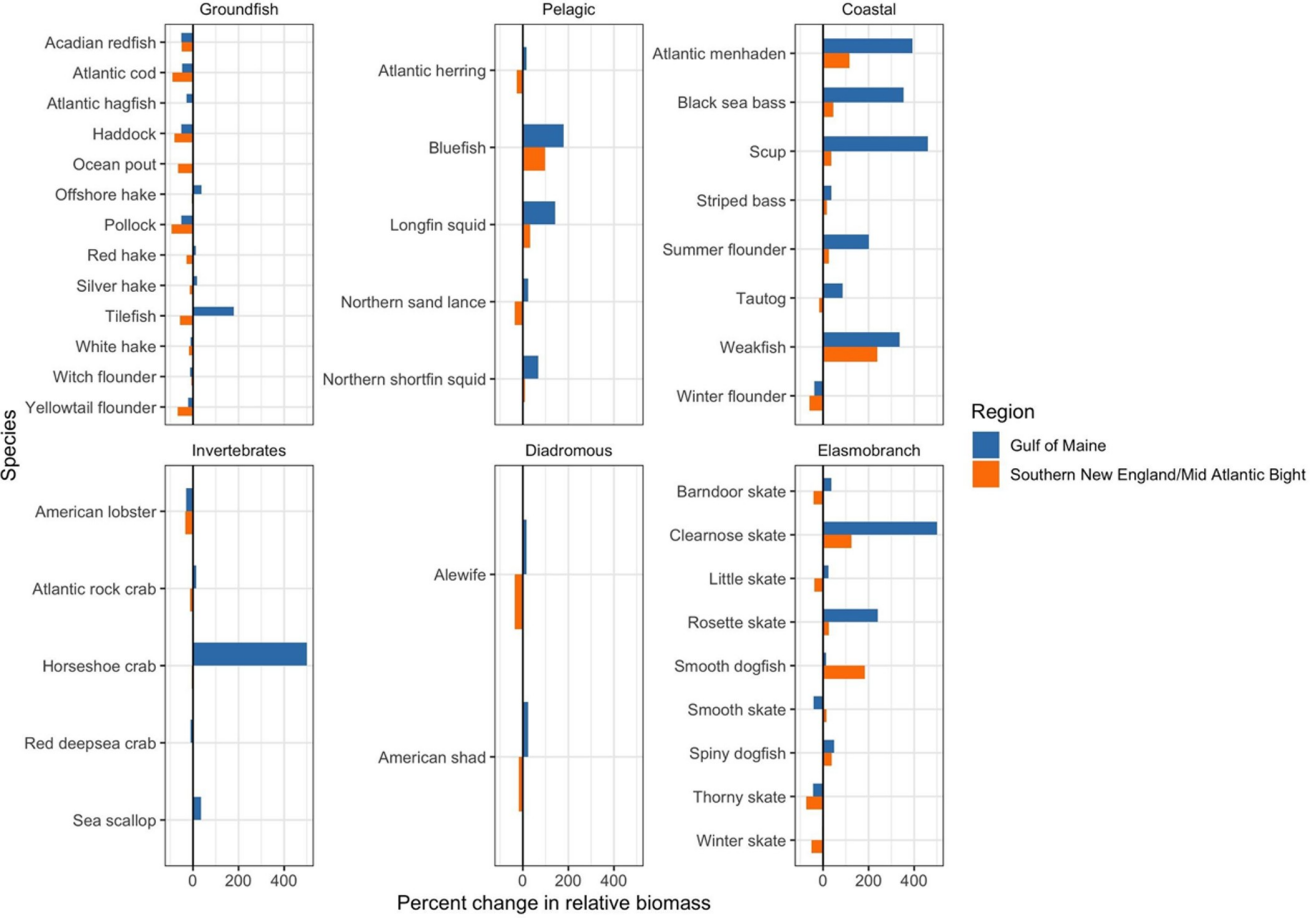
Spring regional projected percent changes in relative biomass by species functional group. Projected species relative biomass changes given the mean CMIP5 RCP 8.5 climate model ensemble projected sea surface temperatures. Positive percent increases were capped to 500% for horseshoe crab and clearnose skate to facilitate visualizing and comparing changes across species and climate model ensemble projected sea surface temperature conditions.

At the large marine ecosystem scale most species showed relatively consistent seasonal patterns (Figs 5 and 6), however, a number of species show opposing seasonal patterns within GoM and SNE-MAB regions (Figs 7 and 8) with projected decreases in the fall and increases in the spring. This fall-decreasing and spring-increasing pattern is most prevalent within the pelagic fish, coastal fish and elasmobranch species groups. Interestingly, none of the projections reveal a fall-increasing and spring-decreasing pattern. Finally, the projected changes in each region appear to be more dramatic in the spring than in the fall, especially for non-groundfish species increasing in the GoM region. For these species, there are noticeable jumps in the projected percent change in relative biomass from the fall to the spring (Figs 7 and 8).

### Comparing species distribution model projections and expert climate vulnerability assessments

**Fig 9:**
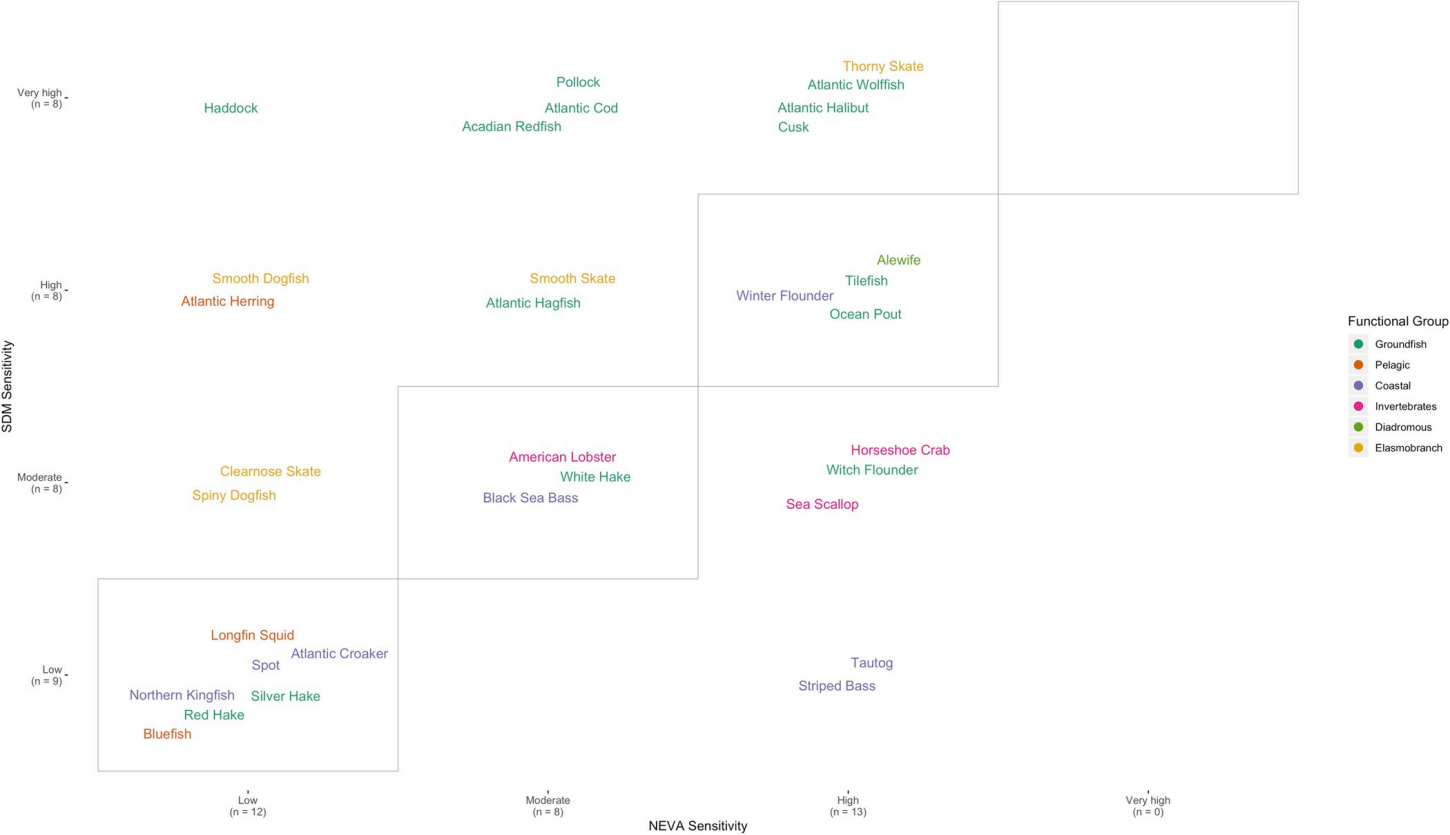
A comparison of sensitivity ranking as determined from the quantitative species distribution model (SDM Sensitivity) and from the qualitative expert vulnerability assessments (NEVA Sensitivity). Bordered diagonal tiles highlight species with matching sensitivity rankings between the two approaches. Along with the species names, the number of species in each sensitivity ranking are shown in parentheses.

**Fig 10:**
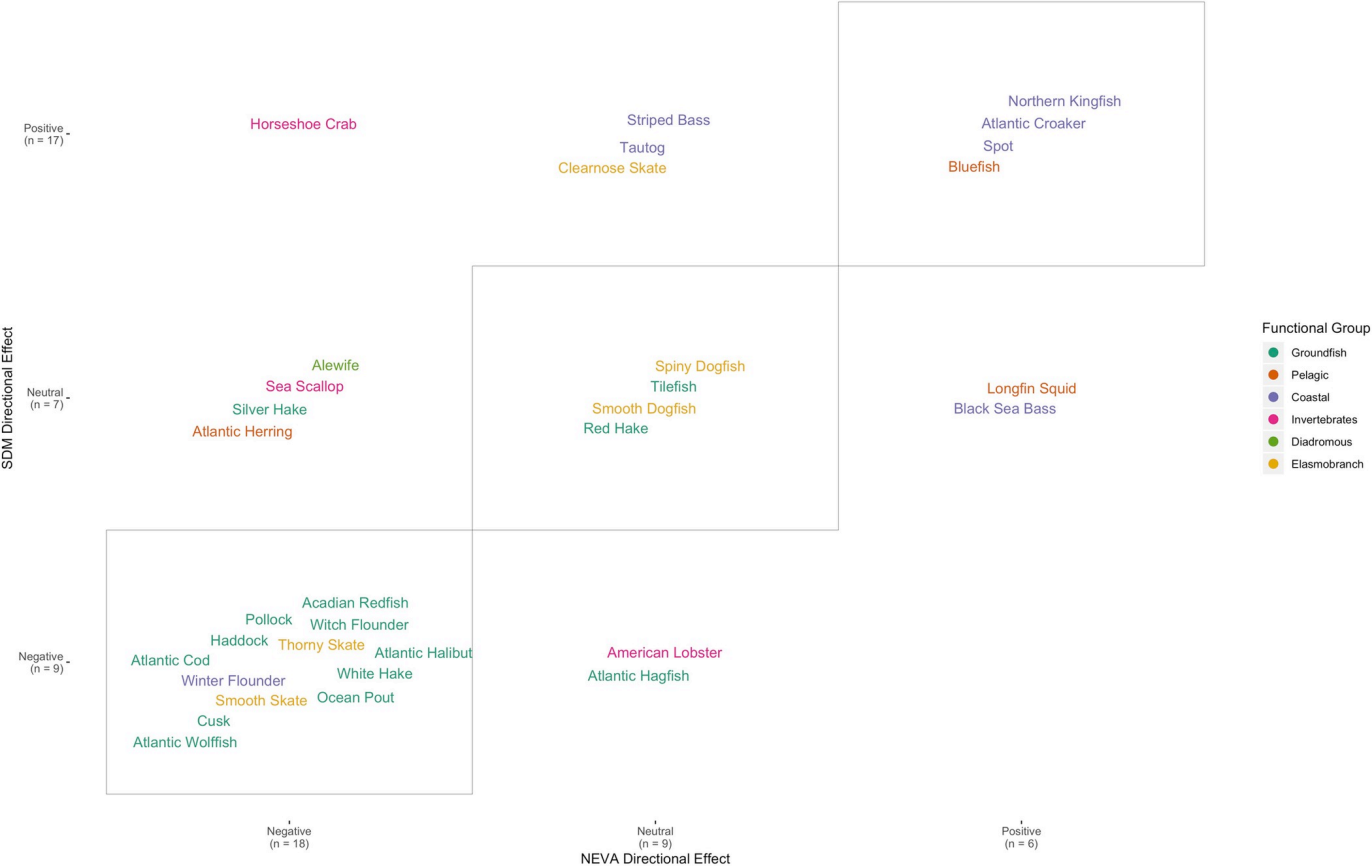
A comparison of directional effect bin assignments as determined from the quantitative species distribution model (SDM directional effect) and from the qualitative expert vulnerability assessments (NEVA directional effect). Bordered diagonal tiles highlight species with matching directional effect classifications between the two approaches. Along with the species names, the number of species in each directional effect bin are shown in parentheses.

For the sensitivity rankings, just under half (14/33) of the species had matching classification ranks (Fig 9). Across the different functional groups, there seemed to be a tendency for the two ranks to match for coastal species, while groundfish sensitivity rankings from the quantitative SDMs were higher for many species than the expert climate vulnerability assessment rankings. A slightly higher matching rate was observed for the directional effect rankings (21/33 species) (Fig 10). Again, matching was relatively common for coastal fish species, while matching occurrences increased among elasmobranch and groundfish species from the low number documented based on sensitivity rankings. Notably, no species fell into the positive NEVA directional effect and negative quantitative SDM directional effect bin.

To further investigate the match/mismatch patterns, we also fit Random Forest classification models to these data, using the species sensitivity attributes as predictor variables. We attempted to do this at the finest scale possible (i.e., each species class was defined by the specific grid cell it was found in Figs 9 and 10). However, both of these models showed relatively poor fits, with out-of-bag classification error rates of 44% for the sensitivity rankings and 38% for the directional effect rankings. Focusing instead on classifying species as either a match or mismatch (i.e., diagonal or off-diagonal in Figs 9 and 10), we were able to fit a model with slightly better classification error rates for the sensitivity component (out-of-bag error rate 28.0%). The model for the directional effect matching was still only barely better than random with an out-of-bag error rate of 35%.

**Fig 11:**
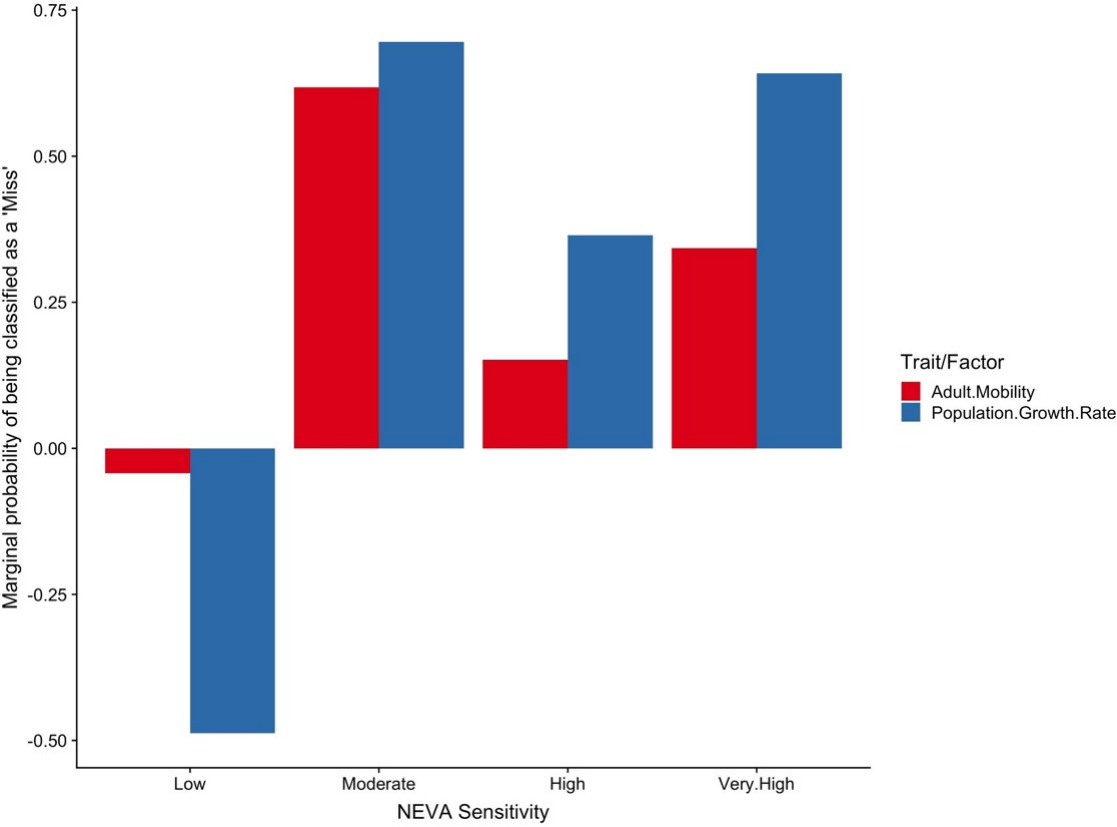
Partial importance plot from fitted Random Forest model classifying match/mismatch in sensitivity ranking between quantitative SDM and qualitative expert assessments. The plot shows the marginal probability of being classified as a “miss” given the NEVA sensitivity ranking for the two most important traits/factors influencing the accuracy of the Random Forest model.

Variable importance plots for the match/mismatch sensitivity model suggest that adult mobility and population growth rate were the two most important factors determining whether sensitivity rankings from the quantitative species distribution model matched the qualitative expert assessments. Marginal effect plots of each of these variables show that species with low sensitivity to each factor had a greater chance of being classified as a match. The chances of a mismatch between classifications increased for species with moderate or higher adult mobility and population growth rate sensitivities (Fig 11).

### Combined species distribution model and expert assessment projections

**Fig 12:**
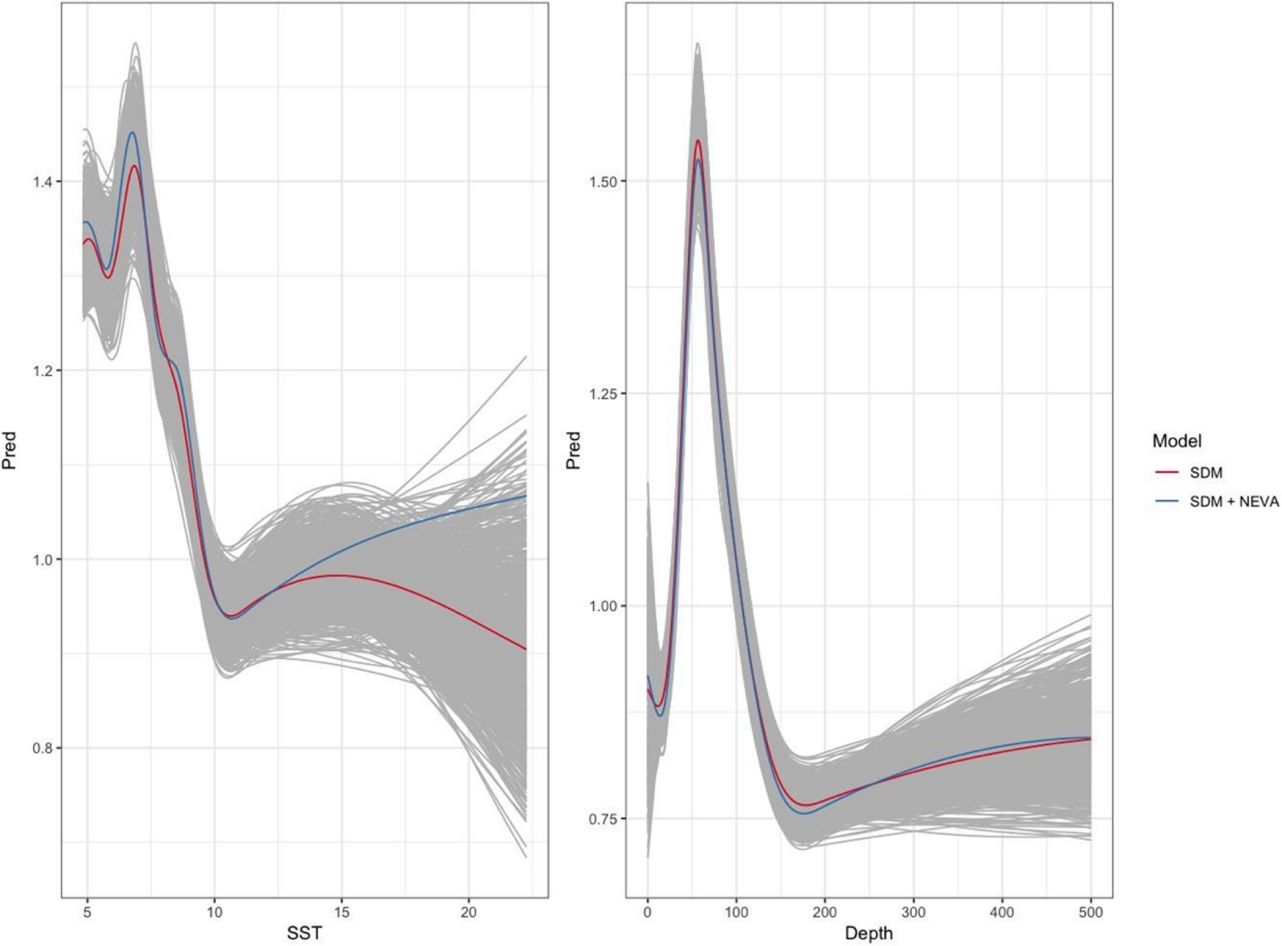
An example comparison between the fitted smooths selected from the combined quantitative species distribution model and qualitative expert assessment data and the fitted smooth from the quantitative species distribution model only. The plot shows predictions for Yellowtail Flounder in the Spring season. Prediction smooth plots were made from the fitted GAM logged positive biomass stage model while varying the variable of interest and holding the other variable constant at its mean. Gray lines show predictions from the suite of candidate models evaluated (n = 1000).

Overall, the combined approach did not influence the prediction accuracy or reduce uncertainty compared to a SDM alone. Using the baseline testing data (2011–2015) to evaluate model predictions, correlation coefficients, standard deviation ratios and RMSE values on average were nearly identical between the two approaches (S3 Table). Although differences between the two methods were evident when looking at the fitted smooth functions selected for each variable (Fig 12), these differences at the parameter scale did not have considerable bearing on the accuracy of species’ relative biomass projections.

## Discussion

### Species distribution model performance

The capacity for SDMs to support forward-looking management and conservation decisions rests on their ability to accurately project species distribution and abundance under future climate conditions. Overall, we were able to successfully fit quantitative SDMs to a number of species (n = 63) across diverse functional groups. Of those, 49 species had models with at least “good” classification ability (AUC > = 0.7), where presences were observed in cells with relatively higher predicted probabilities than absences. While the AUC statistic provides a first assessment of model predictive ability, it is a rank-based statistic and ignores whether predictions actually align with observed values, or the calibration of the model [68]. Placing more emphasis on model calibration as a component to the model validation process will undoubtedly increase opportunities to scrutinize SDM projections as they will be directly compared to observed values, rather than on a relative scale. However, this transition is critical if we want to improve projections and reach a point where they can be trusted and used in decision making processes.

Our work using hold-out testing data to evaluate the predictive skill of SDMs highlights the strength and weaknesses of SDMs across a wide range of species. Correlation coefficients between predicted and observed biomass suggest that while models might be capturing relative patterns and trends, we have considerable work to do to improve SDM projections to accurately represent observed biomass spatial patterns at fine scales. In particular, RMSE values were quite high for most species, and low standard deviation ratio values suggest that model predictions have less spatial variability (i.e., are smoother) than true species distribution and abundance patterns. Including additional environmental variables or using joint species distribution models that incorporate species interactions may increase the ecological realism of SDMs (e.g., 75–77). There may also be opportunities to improve species distribution and abundance projections by including population dynamics with spatial distribution modeling. This call for integration is supported by our findings that adult mobility and population growth rate had the largest effect on whether SDM and qualitative assessment rankings matched. Bridging these two disciplines would leverage our best understanding of population processes that shape the number of individuals and the physical or biological conditions that influence their habitat choices. Such developments will increase model capacity to support climate-smart management and conservation decisions.

### Projected changes in the Northeast Shelf Large Marine Ecosystem

#### Overview

The continued warming of NELME waters will influence the abundance of a diverse suite of marine fish and invertebrate species. Under mean RCP8.5 climate model ensemble SSTs, many species will decline in both seasons, including commercially important groundfish and invertebrate species. Projected decreases for species such as Atlantic herring and northern sand lance are also concerning given the vital role these forage species play in transferring secondary production to higher trophic level species [75]. Coastal fish species were among the only ones to show considerable increases in relative biomass under mean projected SSTs in both seasons. For many of these species, projected increases were greatest with 95^th^ percentile SSTs from the RCP8.5 climate model ensemble. Differences in projected relative biomass changes across climate conditions were also most dramatic for coastal species, especially in the spring season.

Despite analysis differences, our species projections generally align with previous work completed by Kleisner et al. [40]. In their analysis, they also fit two-stage delta generalized additive models to the NOAA NEFSC bottom trawl survey data. However, they included bottom temperature and habitat strata as additional predictor variables, combined data from both seasons to fit the models, and projected SSTs from a higher-resolution global climate model [45]. Though such differences would seem enough to create contrasting patterns, projections across many species show similar patterns. For example, both efforts project consistent spring and fall declines (relative biomass reported here and thermal habitat abundance by Kleisner et al.) for all groundfish species. Differences in species projections did arise for some species in the spring. For example, we project an increase in black sea bass and a decrease in most elasmobranch species while Kleisner et al. projects the opposite patterns [40].

**Fig 13:**
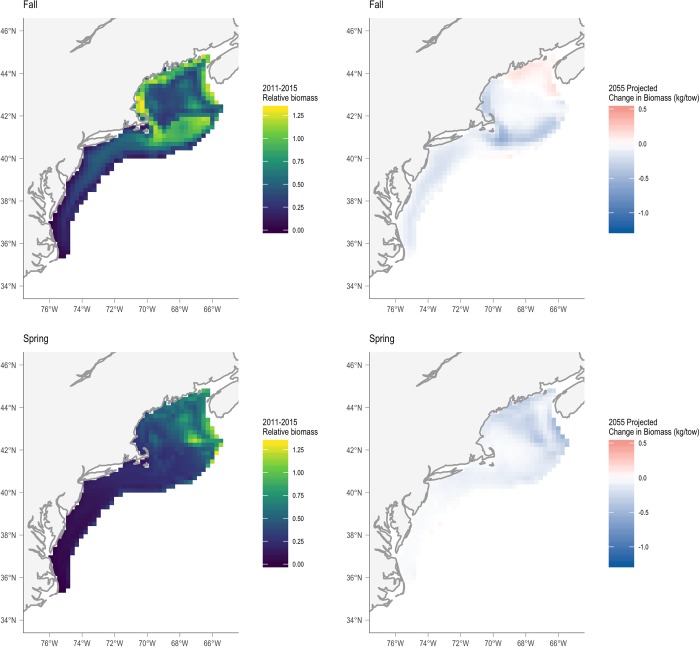
Seasonal average baseline (2011–2015) and future projected (2055) changes in the American lobster distribution and abundance. The maps for the baseline period were created using the seasonal American lobster fitted quantitative SDM and OISST temperature data. The projected change maps were calculated by first projecting lobster distribution and abundance using CMIP-5 ensemble mean temperatures and then calculating the difference between projected and baseline relative biomass values at each grid cell.

In addition to the opposing seasonal projections for some coastal fish and elasmobranch species, our projections for American lobster also diverge. This divergence is especially troubling given that this species currently supports one of the most valuable fisheries in the U.S. [76]. Whereas our projections suggest a modest decrease in lobster biomass across the NELME in both the fall and the spring seasons by 2055 (Fig 13), Kleisner et al. projected large increases in spring (~500%) and fall (~300%) thermal habitat abundance (i.e., density) over the next 60–80 years compared to a baseline period of 1991–2013 [40]. One potential reason for these differences is the season-specific modeling approach we used compared to the year-round approach used by Kleisner et al. The Kleisner et al. approach results in a unimodal lobster-temperature response, while our season-specific approach produces bimodal lobster-temperature responses. In turn, at least for the spring, the Kleisner et al. model will consistently yield higher predicted lobster biomass as SSTs increase because they remain within the range of temperatures used by lobster during the fall season (S1 Fig). In contrast, our model uses data suggesting that lobster are not found in these warmer waters during the spring season. However, this reasoning does not explain divergent projections between the two models in the fall season. Interestingly, Le Bris et al. projected declines in American lobster abundance in both the GoM and SNE using a detailed population dynamics model, which allowed for temperature to influence growth, survival and productivity [77]. Moreover, the overall NEVA sensitivity rank for American lobster was moderate and the expected directional effect was neutral [23]. These competing projections, especially from the SDMs, reinforce the need to understand the sources of uncertainty that contribute to substantial differences and to build collaborations among modelers from multiple disciplines. Ultimately, providing more robust and consistent projections with explanations for divergent model results will be crucial if ecological projections are going to support decision-making processes.

#### Species distribution shifts

While projected changes for the entire NELME are informative, averaging across such a broad and diverse region may smooth over asymmetrical changes within the region. Our investigation of projected relative biomass changes in the GoM and SNE-MAB regions suggests that the distributions of many species are shifting within these regions. For example, groundfish are declining in both regions, however, declines are more severe in the SNE-MAB region. Historically, the southern GoM and SNE-MAB regions marked the southern extent for most of these species’ ranges [78], and projected declines suggest many groundfish distributions will continue to shift towards or beyond the Gulf of Maine. Using observational data, Schuetz et al. also found the distributions of many groundfish species tracked SST changes [7]. However, the influence of different temperature variables and scales varied among species, and similar tracking responses seemed to be more associated with shared habitat use geographies than similarity in life history traits [7].

Along with commercially important groundfish species, many ecologically important species are shifting their distributions in response to warming ocean temperatures. Across both regions and seasons, the biggest changes are projected for the GoM during the spring season. During this period, warmer-water pelagic and coastal species are expected to increase, including forage species such as Atlantic menhaden as well as voracious predators such as bluefish (*Pomatomus saltatrix*) and striped bass. Together these changes could have important implications for the composition and dynamics of the fish community, and they could potentially exacerbate groundfish declines [79].

These projected distribution shifts add to the evidence that the fish community composition within the NELME, and particularly the GoM, is changing. Lucey and Nye (2010), and more recently Kleisner et al. (2016), characterize this shift as one from a “cold-water” assemblage to a “warm-water” assemblage more similar to the fish community found in the SNE-MAB area. Interestingly, relative to the SNE-MAB area, Lucey and Nye (2010) found the GoM fish community to be relatively stable using the same NOAA NEFSC bottom trawl survey data from 1963–2008. In contrast, our work, which includes more recent survey data, suggests that the GoM is expected to experience large changes across species with continued warming ocean temperatures.

There are a few important things to consider related to our findings. Like other single-species SDMs that only include environmental variables, our SDMs do not account for species interactions and other biological processes that may structure a species’ distribution and its capacity to move into new habitat areas. Additionally, they do not account for habitat connectivity, which may also influence a species’ ability to move throughout the ecosystem [4–7]. Focusing within regions, our findings that fewer species are expected to increase in the SNE-MAB than the GoM under future climate conditions are strongly influenced by the data we used to fit the SDMs. In particular, we were able to better model species moving into the GoM than the SNE-MAB because of the geographic area sampled by the NOAA NEFSC bottom trawl survey. To represent movement into the SNE-MAB, we would need data from farther south, such as surveys conducted by the NOAA Southeast Fisheries Science Center. Morley et al. (2018) analyzed distribution changes across survey regions and found that species in the southeast U.S. regions had relatively smaller average projected shifts in centers of distribution than those in the northeast U.S, potentially due to slower warming rates in the southeast U.S. and Gulf of Mexico [46].

### Advancing our projection and forecasting capacity: Comparing and synthesizing quantitative distribution models and qualitative vulnerability assessments

Meeting the challenges arising from species distribution shifts requires accurate and robust models to project species distribution changes under future climate conditions. We worked towards this goal by synthesizing quantitative species distribution models with a qualitative species climate vulnerability assessment in an effort to build off the strengths of each approach. Our results indicated minor changes in the accuracy of combined model projections compared to only the quantitative SDM. This minor influence is likely attributable to our approach, which restricted the candidate models to those generated from the fitted GAM. Although this ensured the species-environment relationships were structured by the quantitative SDM, it also limited the influence of the qualitative assessments. This was especially the case for species with relatively good SDMs since the similarity of candidate model smooths increases as model uncertainty decreases.

Given our findings, we suggest a few alternative approaches to use quantitative and qualitative approaches to increase our ability to accurately project species distributions under future climate change scenarios. For areas where vulnerability assessments have already been completed independently from species distribution modeling efforts, one option would be to use proposal distributions for all GAM components to allow the qualitative assessment data to influence the fitted model parameters and the covariances among parameters that define each GAM smooth rather than sampling from the fully specified smooth functions. Each sample could then be evaluated based on its ability to generate region-wide projections that match the survey data and align with the sensitivity and directional effect of qualitative assessment rankings. In areas where fisheries survey data are available and vulnerability assessments have yet to be completed, we suggest SDMs be incorporated into the vulnerability assessment process such that experts can use this information in their rankings and provide feedback on important processes that species distribution modelers should work to include in SDMs. Another potentially promising way forward would be to expand on the “semi-quantitative” approach proposed by Sortini et al. [37] to also include expert assessments of climate exposure factors other than temperature (e.g., salinity, pH, ocean currents) along with the exposure generated from SDM projections. Finally, integrating information and models across population dynamics, spatial ecology, and general biology will be necessary to improve the quality of information available to meet the ecological, conservation and management challenges arising from climate-driven shifts in species distribution and abundance.

## Supporting information

S1 Table Marine fish and invertebrate species. The full list of species evaluated in the qualitative expert vulnerability assessment completed by Hare et al. (2016), their functional group, and corresponding SDM validation statistics, with SDMs built using data from 1982–2011 and then validated using data from 2011–2015. To be included in our analysis indicating the species distribution model had to have at least “good” predictive ability (AUC > = 0.7) in both seasons when evaluated against the hold out 2011–2015 testing data.
(XLSX)

S2 Table RCP 8.5 CMIP5 climate model ensemble members. A list of the climate model members included in the RCP8.5 CMIP5 ensemble to get projected sea surface temperatures in 2055.
(XLSX)

S3 Table A comparison of model validation statistics between the quantitative species distribution model only and the quantitative species distribution model combined with the qualitative vulnerability assessment. For each statistic, the average across species and seasons was calculated for each combination of “SDM certainty” and “NEVA certainty”. SDM certainty was determined by splitting auc values ranging from 0.7 to 1.0 into five bins of equal width and then assigning low, moderate, high and very high certainty ranks. NEVA certainty was determined by converting sensitivity effect and directional effect certainty values into integers (i.e., low certainty = 1, moderate certainty = 2, high certainty = 3, very high certainty = 4), adding the two values, splitting combined sensitivity and directional effect certainty values ranging from 2 to 8 into five bins of equal width and then assigning low, moderate, high, and very high certainty ranks.
(XLSX)

S1 Doc Projected SST changes in Northeast Shelf Large Marine Ecosystem. A summary and discussion of warming trends under the RCP 8.5 “business as usual” climate model ensemble within the Northeast Shelf Large Marine Ecosystem and the Gulf of Maine (GoM) and Southern New England-Mid Altnatic Bight (MAB) regions.
(DOCX)

S1 Fig American lobster used sea surface temperatures across all seasons, for fall only, and for spring only. Density curves were calculated using NOAA Northeast Fisheries Science Center bi-annual bottom trawl survey data from 1982–2011.
(TIFF)
