# Supplementary material for: Comparing and synthesizing quantitative distribution models and qualitative vulnerability assessments to project marine species distributions under climate change
Source: PLoS One. 2020 Apr 16;15(4):e0231595. doi: 10.1371/journal.pone.0231595 (PMC7161985; doi:10.1371/journal.pone.0231595)
Supplement: S1 Doc — A summary and discussion of warming trends under the RCP 8.5 “business as usual” climate model ensemble within the Northeast Shelf Large Marine Ecosystem and the Gulf of Maine (GoM) and Southern New England-Mid Altnatic Bight (MAB) regions. (DOCX) [file pone.0231595.s004.docx]

## Projected SST changes in Northeast Shelf Large Marine Ecosystem

**Results**

Throughout the NELME, SSTs are expected to continue increasing under the RCP8.5 (“business as usual”) scenario for greenhouse gas emissions over the remainder of the 21st century (Fig S3-1). Warming rates are higher in the fall than in the spring for all of the regions (NELME: fall = 0.039^o^C yr^-1^ and spring = 0.032^o^C yr^-1^; GoM: fall = 0.040^o^C yr^-1^ and spring = 0.036^o^C yr^-1^; SNE-MAB: fall = 0.037^o^C yr^-1^ and spring = 0.028^o^C yr^-1^), with the greatest warming rates projected for the GoM in the fall season and lowest warming rates projected for the SNE-MAB in the spring. Warming rates are more consistent among the regions in the fall, and more variable in the spring, particularly after mid-century (~2050).


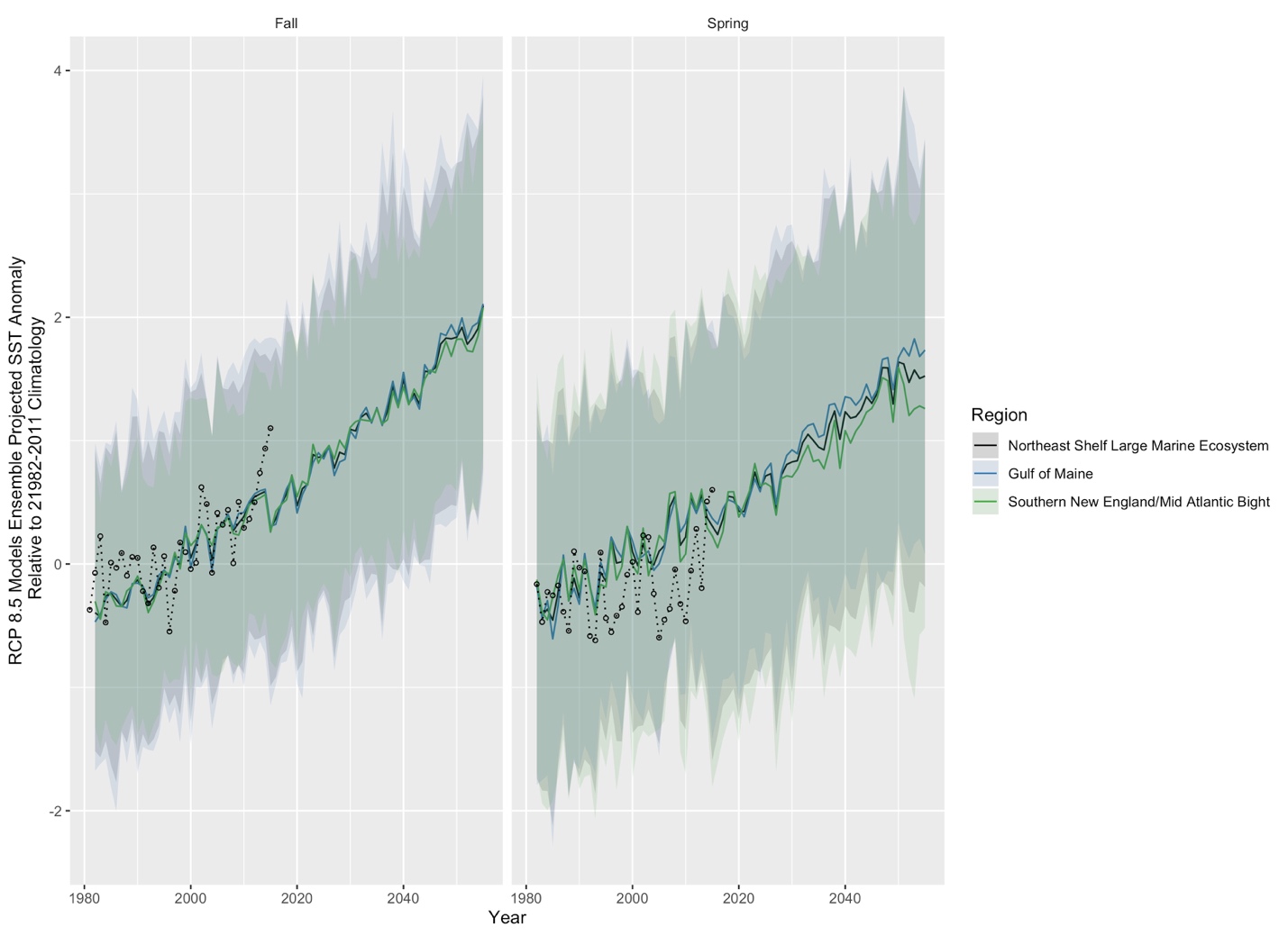


**Fig S3-1. Climate model ensemble projected sea surface temperature anomalies for the Northeast Shelf Large Marine Ecosystem.** The projected sea surface temperature anomalies are calculated using an ensemble of climate models (25 individual members), each run under the RCP8.5 “business as usual” scenario. Average yearly anomalies are shown (mean = solid lines, shaded region = 5th and 95th percentiles) for the entire Northeast Shelf Large Marine Ecosystem, the Gulf of Maine and the southern part of the Northeast Shelf Large Marine Ecosystem, which includes southern New England and Mid-Atlantic Bight. The points overlaid on the climate model ensemble projected sea surface temperature represent observed sea surface temperatures from the NOAA Optimum Interpolation Sea Surface Temperature (OISST) dataset.

**Discussion**

Projections using an ensemble of climate models run under the RCP8.5 “business as usual” scenario suggest that marine resources in the NELME will undergo considerable changes in the future. This large marine ecosystem has already been warming rapidly [1,2], and projections indicate substantial future increases in SST, with some seasonal differences as the warming rate is slightly faster in the fall than in the spring. These patterns agree with previous SST analyses, which used historical observations [3] and climate model projections [4,5]. In their analysis using historical SST, Thomas at el. (2017) also noted spatial and seasonal patterns in warming rates within the region [3]. Our results suggest that this asymmetry will persist under future climate conditions as warming rates for the GoM were slightly greater during the fall and substantially greater during the spring than in the SNE-MAB. Kleisner et al. (2017) reported similar spatial variability in warming rates between the SNE-MAB and the GoM using projected SSTs from a higher resolution global climate model (CM2.6) that better resolves the position and influence of the Gulf Stream on the NELME [43]. Rather than a specific RCP driving emission rates, this model was driven by increasing CO_2_ concentrations 1% per year for 80 years [6]. The final 20 years of the simulation roughly correspond to years 2060-2080 of RCP8.5 climate model scenarios [4,6]. While patterns were similar, projected warming rates from CM2.6 were slightly greater than the CMIP5 ensemble under the RCP8.5 scenario for every region and season.

**References**

1. Pershing AJ, Alexander MA, Christina M, Kerr LA, Bris A Le, Mills KE, et al. Slow adaptation in the face of rapid warming leads to collapse of the Gulf of Maine cod fishery. Science. 2015;350: 809–812. doi:10.1126/science.aac9819

2. Pershing AJ, Mills KE, Dayton AM, Franklin BS, Kennedy BT. Evidence for adaptation from the 2016 marine heat wave in the northwest Atlantic ocean. Oceanography. 2018;31: 152–161. doi:10.2307/26542661

3. Thomas AC, Pershing AJ, Friedland KD, Nye JA, Mills KE, Alexander MA, et al. Seasonal trends and phenology shifts in sea surface temperature on the North American northeastern continental shelf. Elementa: Science of the Anthropocene. 2017;5. doi:10.1525/elementa.240

4. Kleisner KM, Fogarty MJ, Mcgee S, Hare JA, Moret S, Perretti CT, et al. Marine species distribution shifts on the U.S. Northeast Continental Shelf under continued ocean warming. Progress in Oceanography. 2017;153: 24–36. doi:10.1016/j.pocean.2017.04.001

5. Alexander MA, Scott JD, Friedland KD, Mills KE, Nye JA, Pershing AJ, et al. Projected sea surface temperatures over the 21st century: Changes in the mean, variability and extremes for large marine ecosystem regions of Northern Oceans. Elementa: Science of the Anthropocene. 2018;6: 9. doi:10.1525/elementa.191

6. Saba VS, Griffies SM, Anderson WG, Winton M, Alexander MA, Delworth TL, et al. Enhanced warming of the Northwest Atlantic Ocean under climate change. Journal of Geophysical Research: Oceans. 2016;121: 118–132. doi:10.1002/2015JC011346
