# Supplementary figures and images for: Comparing and synthesizing quantitative distribution models and qualitative vulnerability assessments to project marine species distributions under climate change
Source: PLoS One. 2020 Apr 16;15(4):e0231595. doi: 10.1371/journal.pone.0231595 (PMC7161985; doi:10.1371/journal.pone.0231595)

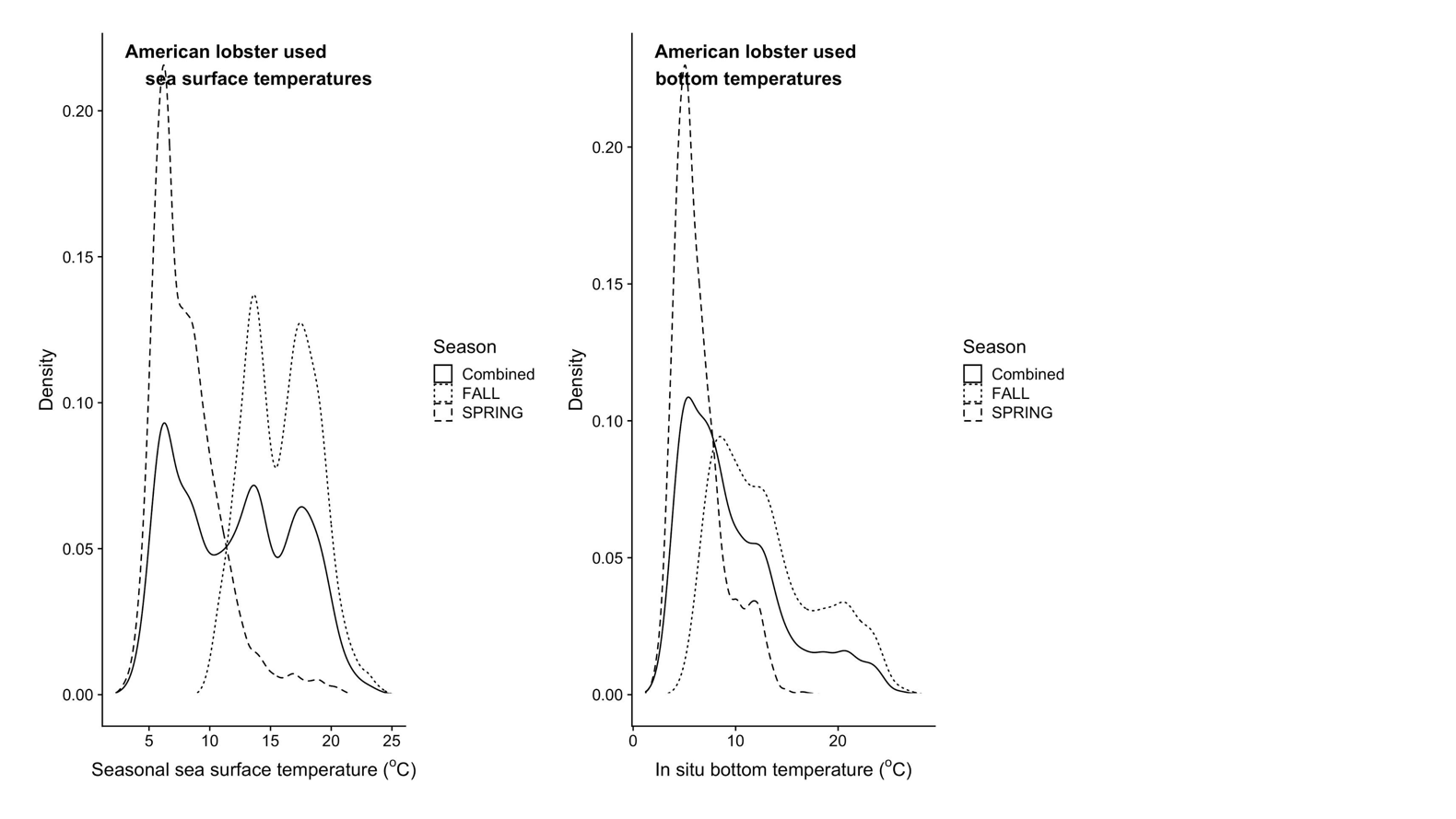

Supplement: S1 Fig — Density curves were calculated using NOAA Northeast Fisheries Science Center bi-annual bottom trawl survey data from 1982–2011. (TIFF) [file pone.0231595.s005.tiff]
